# Ferroptosis Mediation Patterns Reveal Novel Tool to Implicate Immunotherapy and Multi-Omics Characteristics in Bladder Cancer

**DOI:** 10.3389/fcell.2022.791630

**Published:** 2022-01-25

**Authors:** Jingchao Liu, Zhipeng Zhang, Wei Zhang, Lingfeng Meng, Jiawen Wang, Zhengtong Lv, Haoran Xia, Meng Wu, Yaoguang Zhang, Jianye Wang

**Affiliations:** ^1^ Department of Urology, Beijing Hospital, National Center of Gerontology, Institute of Geriatric Medicine, Chinese Academy of Medical Sciences, Beijing, China; ^2^ Graduate School of Peking Union Medical College, Chinese Academy of Medical Sciences, Beijing, China

**Keywords:** bladder cancer, ferroptosis, immunotherapy, polymerase Chain Reaction, tumour mutation burden

## Abstract

**Background:** The regulatory role of ferroptosis in malignant tumours has been recently demonstrated. However, the potential roles of ferroptosis mediation patterns in bladder cancer remain elusive.

**Materials and Methods:** The ferroptosis mediation patterns of 889 bladder cancer samples were comprehensively evaluated based on ferroptosis-related genes. The underlying correlations between these mediation patterns and multi-omic characteristics of bladder cancer were systematically analysed. The ferroptosis mediation patterns of individual samples were quantified by ferropscore using the principal component analysis algorithm. The typical ferroptosis-related genes with prognostic roles were further randomly validated using immunohistochemical staining, real-time polymerase chain reaction and western blotting.

**Results:** Three different ferroptosis mediation patterns were identified. The abundance of infiltration of 23 immune cells was different among the three mediation patterns. The quantification of ferroptosis mediation patterns in individual samples served as a promising tool for predicting patient survival outcomes; immune cell infiltration abundance; tumour mutation burden; oncogenic mutation status and tumour grade, stage and molecular subtypes. Low ferropscore combined with high tumour mutation burden was associated with the best survival prognosis. Expressions of PD-L1 (*p* < 0.001), PD-1 (*p* = 0.002) and CTLA-4 (*p* = 0.003) were all significantly upregulated in the high ferropscore group. Low ferropscores also predicted good immunotherapy response for anti-CTLA4 strategy. The mRNA and protein levels of FADS2, a typical ferroptosis-related gene used in the study, were higher in bladder cancer cell lines than in controlled SV-HUC-1 cells. In addition, immunohistochemical staining revealed significantly higher expression levels of FADS2 in human bladder cancer tumour tissues than in normal tissues.

**Conclusion:** This study identified three distinct ferroptosis mediation patterns in bladder cancer. Quantification of ferroptosis mediation patterns in individual samples may help to improve the understanding of multiomic characteristics and guide future immunotherapy responses to bladder cancer.

## Introduction

Cell death plays an essential role during the development and evolution of multicellular organisms. The progress of tumour treatments at different stages is always synchronised with human cognition for different modes of cell death. In the last decade, increasing studies have focussed on a new mode of cell death—ferroptosis. Ferroptosis was initially discovered as an iron-dependent and lipid reactive oxygen species-accumulated mode of cellular death in 2012. (Dixon et al., 2012). It was distinct from previously known cell death modes including apoptosis, necrosis and autophagy in both cell function and morphological characteristics. Its unique morphological characteristics include the increased density of mitochondrial membrane, shrinkage of cell mitochondria and disappearance of mitochondrial cristae. In ferroptotic cells, cell membrane integrity is retained, the nucleus is normal in size and chromatin is not concentrated, these characteristics are different from those of apoptosis and autophagy. (Xie et al., 2016). Increasing studies have demonstrated that ferroptosis plays a vital role in regulating malignant cell development, and targeted interference of ferroptosis is a promising strategy for future cancer treatment. (Basuli et al., 2017; Chen et al., 2017; Belavgeni et al., 2019). For example, phenylethyl isothiocyanate combined with cotylenin A was reported to induce ferroptosis and inhibit pancreatic cancer development. (Eling et al., 2015) (Louandre et al., 2015). recently discovered that sorafenib played a therapeutic role by inducing ferroptosis in hepatocellular carcinoma. (Louandre et al., 2015). Ferroptosis may also be induced by blocking the MUC1-C signalling pathway in breast cancer, which inhibits cancer cell proliferation. (Ishimoto et al., 2011; Hasegawa et al., 2016). Previous studies have indicated that ferroptosis offers potential prospects for tumour treatment and hence provides a new direction for investigating new anti-tumour methods. (Guo et al., 2018).

Bladder cancer is one of the most common cancers threatening human health and is reported to cause >200,000 deaths worldwide. (Richters et al., 2020). Although bladder cancer can be treated surgically, patients may experience relapse and develop metastasis. The clinical stages or pathological grades are currently used to guide treatment and predict survival outcomes in clinical settings. The typical oncogenic mutations of P53, ERCC2 and RB1 are reported to be responsible for bladder cancer development. (Montie, 2005). Patients diagnosed with advanced bladder cancer are recommended to receive cisplatin-based chemotherapy as the first-line treatment and occasionally immunotherapy as the second-line treatment. (Rouanne et al., 2018). However, owing to the lack of in-depth understanding of mechanisms underlying bladder cancer occurrence, the therapeutic effects of chemotherapy and immunotherapy are only effective in limited patients. Whether ferroptosis plays a role in bladder cancer and is a promising candidate for developing novel treatment strategies remains elusive. This study aimed to comprehensively analyse ferroptosis mediation patterns depending on transcriptome and clinical information of 889 bladder cancer samples and to analyse the inherent correlation between these patterns and various characteristics of bladder cancer. We identified three different ferroptosis mediation patterns that were revealed to have a significant relationship with multiomic characteristics of bladder cancer. Therefore, we quantified the three patterns based on ferropscore to further examine their role in predicting survival outcomes and analysed various clinical characteristics of bladder cancer.

## Materials and Methods

### Bladder Tissue Source, Public Sequencing Dataset and Processing

The paired samples of bladder tissue resections were obtained from patients diagnosed with bladder cancer who received partial cystectomy or radical cystectomy at Beijing Hospital in 2020. The collected tissue specimens were immediately embedded into paraffin for further analysis. Immunohistochemical (IHC) staining was used to quantify the expression levels of typical ferroptosis-related proteins. Public transcriptome data and their corresponding clinical information were downloaded from The Cancer Genome Atlas (TCGA) and the Gene-Expression Omnibus (GEO) databases. Datasets without clinical information were excluded from further analysis. Eventually, four bladder cancer cohorts were included in this study, including TCGA, GSE13507, GSE32894 and GSE31684. Background adjustment was performed using the “affy” and “simpleaffy” R packages for downloaded matrix from the Affymetrix platform. The normalized sources were directly downloaded if the GEO matrix was from the other platforms. The TCGA sequencing matrix was downloaded from https://portal.gdc.cancer.gov/in a FPKM format and translated into TPM values for further analysis. These normalized procedures were aimed to keep consistency and comparability among different TCGA and GEO cohorts in this study. The somatic mutation data and copy number variation (CNV) files were only searched on TCGA cohort.

### Unsupervised Clustering of Ferroptosis-Related Genes in Merged Cohorts

The four bladder cancer cohorts (TCGA, GSE13507, GSE32894 and GSE31684) were merged into a matrix, and 23 ferroptosis-related genes were identified from the merged matrix document to identify distinct ferroptosis mediation patterns in bladder cancer, including ACSL4, ALOX12, CBS, CHAC1, CS, GCLM, GLS2, GSS, HMGCR, MT1G, PTGS2, SAT1, TFRC, HSBP1, ACO1, FTH1, NFS1, ACACA, PEBP1, SQLE, FADS2, ABCC1 and SLC1A5. The prognostic roles of these genes were analysed by univariate cox regression analysis. Subsequently, ferroptosis mediation patterns were analysed by unsupervised clustering analysis based on these genes. The consensus clustering algorithm was used to determine the stability and number of distinct clusters using the “ConsensusClusterPlus” R package. We repeated the process 1,000 times to enhance the classification stability and also performed Kaplan-Meier analysis to analyse survival characteristics of the clusters.

### Gene Set Variation Analysis (GSVA), Gene Ontology (GO) and Single-Sample Gene Set Enrichment Analysis (ssGSEA)

The GSVA was a popular bioinformatics algorithm, which was extensively utilized in cancer-related studies. (Liu et al., 2021a; Liu et al., 2021b; Liu et al., 2021c; Wang et al., 2021; Zhang Y et al., 2021). GSVA was performed to examine different biological activities among the three ferroptosis mediation patterns using the “GSVA” R package. The “c2. cp.kegg.v6.2.-symbols” was referenced to perform GSVA, and significance was set at *p*-value < 0.001. Gene ontology (GO) analysis was performed using the “clusterProfiler” package based on differentially expressed genes (DEGs) among the ferroptosis mediation patterns, defining adjusted *p*-value < 0.01 and |logFC| >1. The single-sample get set enrichment analysis (ssGSEA) was performed to compare the infiltration levels of immune cells among ferroptosis mediation patterns. (Charoentong et al., 2017).

### Quantification of Ferroptosis Mediation Patterns Using Ferropscore

Principal component analysis (PCA) was used to quantify the ferroptosis mediation patterns of individual bladder cancer samples. A ferroptosis mediation signature was developed using the PCA algorithm, which was termed ferropscore.

The detailed scoring procedure is described as follows: DEGs among the three ferroptosis mediation patterns were subjected to univariate Cox regression analysis (*p* < 0.05). Subsequently, the consensus clustering algorithm was used to group all samples into different clusters based on DEGs with a prognostic value, and these groups were named gene clusters. The rationality and significance of gene clusters were evaluated through survival analysis. All DEGs with a prognostic value were further subjected to PCA analysis to develop a ferroptosis mediation signature. We simultaneously input the principal element 1 and 2 into the scoring procedure to enhance the weight of well-correlated ferroptosis-related genes in our set. An algorithm was developed to calculate ferropscore for quantifying ferroptosis mediation patterns of individual samples: ferropscore = ∑ (principal element 1_e_ + principal element 2_e_), where e indicated the expression levels of ferroptosis-related genes. (Zeng et al., 2019). All included samples were then classified into a high- or low-risk group based on the corresponding median ferropscore value.

### Correlation Between Ferroptosis Gene Signature and Multiomic Characteristics

The Kaplan-Meier analysis was initially performed to analyse the role of the ferroptosis risk signature in predicting survival outcomes. Differences in ferropscore between different ferroptosis mediation patterns and gene clusters were compared using the “limma” and “ggpubr” R packages. The “corrplot” package was used to analyse the correlation between ferropscore and infiltration of various immune cells based on ssGSEA analysis. Previous studies have confirmed a close relationship between bladder cancer and various oncogenic mutations including p53, ERCC2, FGFR3, and RB1. (Robertson et al., 2017). The relationship among the ferroptosis signature, oncogenic mutations and tumour mutation burden (TMB) was further analysed using the “ggplot2” and “ggpubr” packages. Concerning the recently reported molecular types of bladder cancer, (Robertson et al., 2017), the relationship between the ferroptosis signature and ‘basal’ or ‘luminal’ molecular subtype was also investigated. Furthermore, the correlation of the ferroptosis signature with various clinical parameters including age, gender and clinical stages was also evaluated using the “ggplot2” package. The potential prognostic role of the ferroptosis signature in predicting survival outcomes was further validated in different sample groups categorised as “age >65 years/age ≤ 65 years”, “Ta–T1/T2–T4”, “female/male” and’G1/G2/G3′ subgroups.

### Potential Guidance for Immunotherapy Response Using the Ferroptosis Signature

First, the differential expression of immune checkpoint blockade genes including PD-L1, PD-1 and CTLA-4 between the high and low ferropscore groups was evaluated using the “limma” package. Furthermore, an immunotherapeutic cohort derived from http://tcia.at/was used to evaluate relationships between the immunotherapeutic scores and ferroptosis signature. The role of the signature in predicting treatment effects of four immunotherapy strategies including anti-CTLA4 alone, anti-PD1 alone, no medication and combined medication was evaluated in the high and low ferropscore groups. The tumour immune dysfunction and exclusion (TIDE) algorithm was further used to analyse the predictive ability of the ferroptosis signature to determine response to immunotherapy in TCGA cohort. (Jiang et al., 2018).

### Cell Lines

The SV-HUC-1 (CL-0222), HT-1376 (CL-0672), BIU-87 (CL-0035), T24 (CL-0227), RT4 (CL-0431) and 5,637 (CL-0002) cell lines were purchased from Procell Life Science and Technology Co., Ltd., Wuhan, China. The RT-112 (C6779) cell was purchased from Beyotime. SV-HUC-1 cells were cultured in Ham’s F-12K medium supplemented with 10% foetal bovine serum (FBS, Gibco); HT-1376 cells were cultured in MEM supplemented with 10% FBS; BIU-87, T24, RT4, RT-112 and 5,637 cells were cultured in RPMI-1640 supplemented with 10% FBS.

### Quantitative PCR

Cells in the logarithmic growth phase were seeded at a density of 5 ×10^5^ cells/well in a 6-well plate and incubated for 48 h. Total RNA was extracted using TRIzol (Invitrogen) according to the manufacturer’s instructions. Complementary DNA (cDNA) synthesised using a cDNA reverse transcription kit (AGbio) and total mRNA templates. The relative mRNA levels of FADS2, HMGCR and GAPDH were quantified via real-time polymerase chain reaction (qPCR) using the SYBR Green Premix Pro Taq HS qPCR kit (AGbio) on a qPCR instrument (Bio-Rad). The mRNA expression levels were normalised to those of GAPDH. All reactions were performed in triplicates.

### Western Blot

Cells in the logarithmic growth phase were seeded at a density of 5 ×10^6^ cells/well in a 6-well plate and incubated for 48 h. The cells were collected and lysed with radio-immunoprecipitation assay (RIPA) lysis buffer supplemented with protease and phosphatase inhibitors on ice for 30 min. The samples were subjected to sodium dodecyl sulfate–polyacrylamide gel electrophoresis (SDS-PAGE; 10% SDS), and the gel was transferred onto polyvinylidene difluoride (PVDF) membranes for western blot analysis. The following antibodies were used for the detection of proteins: rabbit anti-FADS2 (1:1000, Abcam) and mouse anti-HMGCR (1:1000, Abcam. Mouse anti-GAPDH (1:5000, Abcam) was used as a loading control. Proteins were visualised using anti-mouse or anti-rabbit HRP-conjugated secondary antibodies (1:5000, Zhongshan Jinqiao biotechnology company) and ECL-Plus (Millipore). The resulting bands were analysed and quantified using the ImageJ^®^ 1.49 g software (National Institutes of Health, Bethesda, MD, United States). Each experiment was repeated in triplicates.

### Gene Knocking Down Assay

HT-1376 cells were plated at low confluency (30%) in 6-well plates and transfected with constructed shRNA targeted for FADS2 lentivirus, or the control shRNA for GFP lentivirus using polybrene (5 μg/ml) for 4 h. Cells were then grown in fresh media for 24 h. Transfected cell populations were then selected using puromycin (5 μg/ml) for another 24 h. The FADS2 targeting sequences of shRNA is CCC​ACA​TCA​TCG​TCA​TGG​AAA.

### Colony Formation Assay

Two thousand shFADS2 HT-1376 cells and shNC HT-1376 cells were plated per well in 6-well plates and incubated for 72 h. Refresh the media every 3 days for about 2 weeks. After removal of the culture media, cells were washed by PBS twice and colonies were fixed by methanol for 10 min. Cells were then stained with 0.1% crystal violet for 10 min. Plates were imaged using a camera. Three independent experiments were performed.

### Immunohistochemical Staining

IHC staining was performed as described in a previous study. (Ertao et al., 2016). Briefly, 10-mm sodium citrate buffer was used to pretreat deparaffinised sections for antigen unmasking. The sections were blocked with normal serum and incubated with primary antibodies at 4°C overnight. The following day, the sections were incubated with the secondary antibody after rinsing. The VECTASTAIN ABC kit was used to amplify signals according to the manufacturer’s instructions. Diaminobenzidine was used as the substrate to visualise proteins. A semi-quantitative strategy was adopted to quantify the FADS2 staining intensity based on four scoring grades as follows: “0, negative”; “1, weak”; “2, moderate” and “3, strong”. Two pathologists unaware of the clinical information of corresponding specimens performed the analysis independently.

### Statistical Analysis

The Student’s t-test was used to identify DEGs between different groups. The categorical variables were compared using the chi-squared test. The Mann–Whitney test was performed to compare ssGSEA scores among different groups. Differences among three or more groups were compared using one-way analysis of variance (ANOVA). The Kaplan-Meier analysis with the log-rank test was conducted to compare the survival outcomes of different ferroptosis mediation patterns. The R, version 4.0.5 (Vienna, Austria) or IBM SPSS Statistics for Windows, version 23.0 (IBM Corp., Armonk, NY, United States) was used to perform relative statistical and analytical analyses.

## Results

### Genetic Variation Landscape of Ferroptosis-Related Genes in Bladder Cancer

**FIGURE 1:**
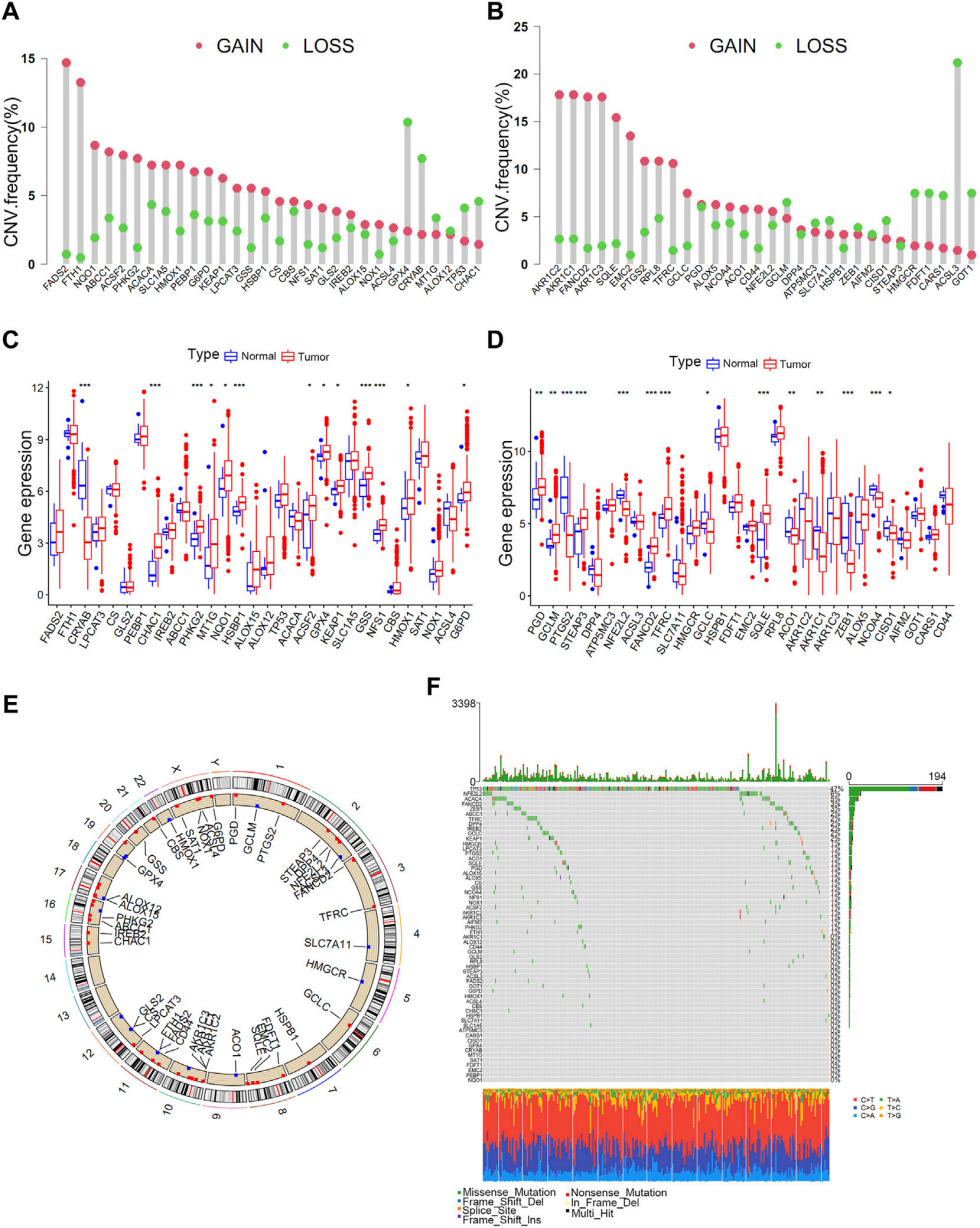
Landscape of genetic variations in ferroptosis-related genes in bladder tumours. **(A)** CNV distributions of ferroptosis-related genes in bladder tumours. **(B)** CNV distributions of ferroptosis-related genes in bladder tumours. **(C)** Different expressions of ferroptosis-related genes in tumour and normal tissues. **(D)** Different expressions of ferroptosis-related genes in tumour and normal tissues. **(E)** CNV locations of ferroptosis-related genes on 23 human chromosomes. **(F)** Waterfall plot demonstrating the somatic mutation status of ferroptosis-related genes in bladder cancer. Each column indicates individual samples, and the upper bar plot represents the TMB value. The number on the right represents the frequency of somatic mutations.

High-quality studies on the topic of ferroptosis in the last 5 years were systematically reviewed to identify ferroptosis-related genes in this study. The searched high-quality researches on ferroptosis were defined as novel studies with impact factors >10 points and a total of 60 ferroptosis-related genes were included in this study. (Stockwell et al., 2017; Bersuker et al., 2019; Doll et al., 2019; Hassannia et al., 2019). All genes are listed in Supplementary Table S1. The copy number variation (CNV) (Supplementary Table S2) and somatic mutations (Supplementary Table S3) of ferroptosis-related genes were investigated in TCGA cohort. CNV analysis revealed a significant CNV occurrence frequency of ferroptosis-related genes (Figure 1A,B). Most genes were characterised by copy number amplification, including FADS2, FTH1, NQO1, ABCC1, AKRIC2, AKRIC1, FANCD2, and AKR1C3, whereas some genes including ACSL3, GOT1, FDFT1, HMGCR, GPX4, and CRYAB were characterised by copy number deletion in bladder cancer. Most ferroptosis-related genes exhibited significantly different expressions between bladder tumour tissues and normal tissues (Figures 1C,D). Furthermore, ferroptosis-related genes with copy number amplification (including FADS2, NQO1, ABCC1, ACSF2, HMOX1, FANCD2 and SQLE) demonstrated higher expression in tumour tissues, and those with copy number deletion (including CRYAB, ACSL3, ZEB1) demonstrated lower expression in tumour tissues. Figure 1E demonstrates detailed locations of CNVs in ferroptosis-related genes on chromosomes. As shown in Figure 1F, 262 out of 412 samples (63.59%) have mutations in ferroptosis-related genes. The high heterogeneity of copy number and genetic mutation indicated important roles of ferroptosis-related genes in bladder cancer development.

### Ferroptosis Mediation Patterns Depending on 23 Ferroptosis-Related Genes

**FIGURE 2:**
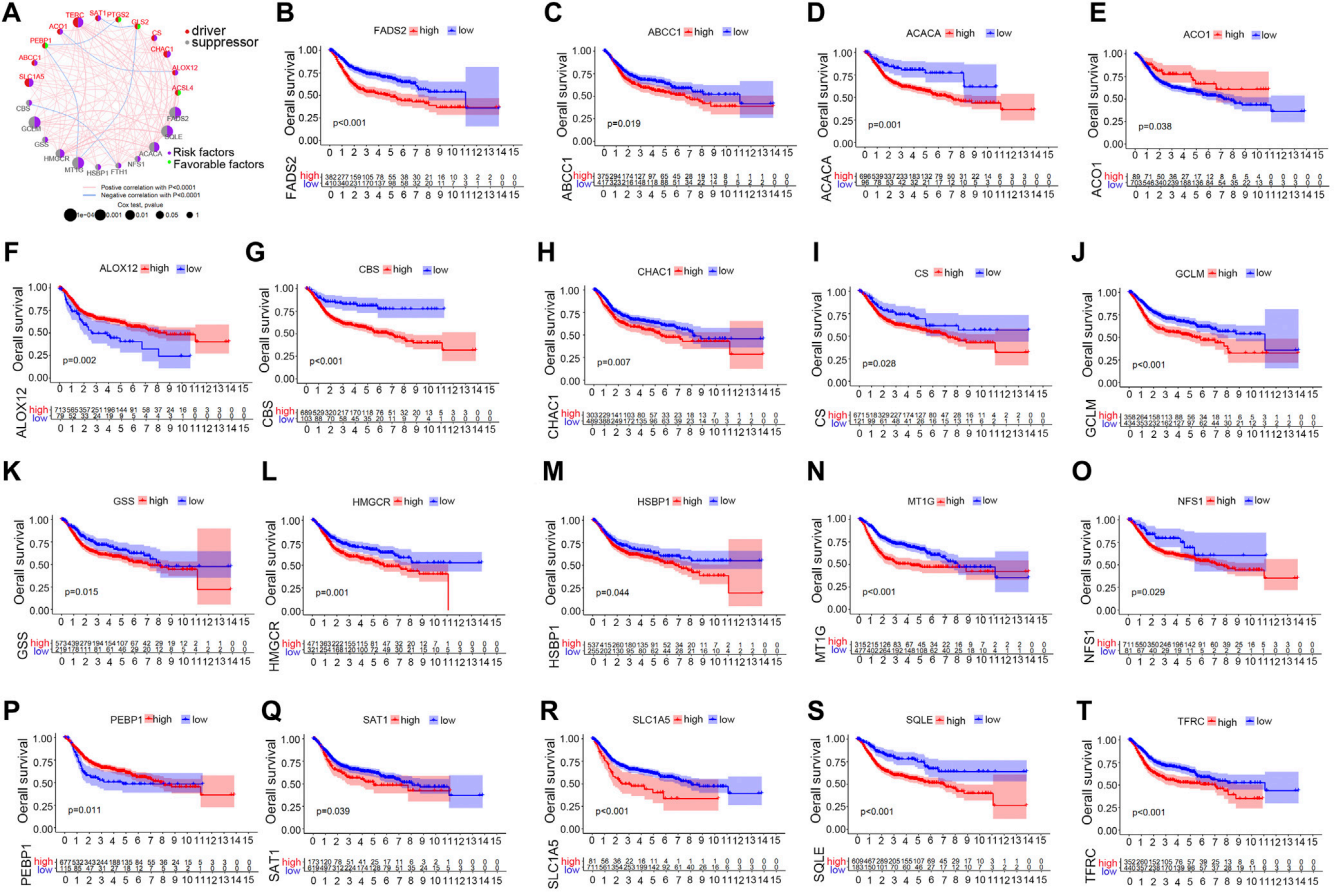
The prognostic role of ferroptosis-related genes in the merged cohort. **(A)** A network illustrating interactions between 23 ferroptosis-related genes. **(B–T)** Kaplan-Meier analysis of 19 ferroptosis-related genes with prognostic roles in bladder cancer in the merged cohort.

The TCGA, GSE13507, GSE32894 and GSE31684 cohorts were merged into a single matrix document to conduct further analysis (Supplementary Table S4). Because these cohorts belonged to distinct sequencing platforms, only 23 ferroptosis-related genes were derived from the merged cohort including 889 bladder cancer samples (Supplementary Table S5). We first analysed the interactions among these 23 genes and further investigated their prognostic value in predicting survival outcomes. Figure 2A demonstrated a close relationship among the 23 ferroptosis-related genes. FADS2, TFRC, GCLM and MTIG acted as key factors in the core of the relational network. We discovered that 19 out of 23 genes with a frequency of 82.6% had significant prognostic value for predicting bladder cancer survival outcomes. (Table 1). These genes included FADS2, ABCC1, ACACA, ACO1, ALOX12, CBS, CHAC1, CS, GCLM, GSS, HMGCR, HSBP1, MT1G, NSF1, PEBP1, SAT1, SLC1A5, SQLE and TFRC. Kaplan-Meier analysis of these prognostic genes was shown in Figures 2B–T. These results indicated that all ferroptosis-related genes interacted with each other to play vital roles in bladder cancer development. Furthermore, all merged samples were classified into three distinct ferroptosis mediation patterns depending on the expression levels of the 23 ferroptosis-related genes using the “ConsensusClusterPlus” R package (Supplementary Figure S1A). Detailed cluster results of merged samples are listed in Supplementary Table S6. As shown in Supplementary Figure S1B, Ferropcluster A could predict significantly-improved survival outcomes. The heatmap in Supplementary Figure S1C demonstrated a detailed relationship between ferropclusters and various clinical characteristics.

**TABLE 1:** Prognostic analysis of ferroptosis-related genes for survival outcomes in merged cohort.

Gene ID	HR	HR.95L	HR.95H	*p*-value
ACSL4	0.92	0.76	1.10	0.06
ALOX12	0.99	0.90	1.11	**< 0.01**
CBS	1.07	0.95	1.21	**< 0.01**
CHAC1	1.21	1.04	1.42	**< 0.01**
CS	1.16	0.91	1.49	**0.03**
GCLM	1.28	1.14	1.44	**< 0.01**
GLS2	0.88	0.67	1.17	0.11
GSS	1.24	0.99	1.54	**0.01**
HMGCR	1.30	1.08	1.56	**< 0.01**
MT1G	1.15	1.07	1.23	**< 0.01**
PTGS2	0.99	0.92	1.07	0.07
SAT1	1.05	0.92	1.20	**0.04**
TFRC	1.28	1.14	1.44	**< 0.01**
HSBP1	1.10	0.89	1.36	**0.04**
ACO1	1.06	0.90	1.24	**0.04**
FTH1	1.14	0.97	1.33	0.06
NFS1	1.08	0.82	1.42	**0.03**
ACACA	1.40	1.15	1.70	**< 0.01**
PEBP1	0.92	0.77	1.10	**0.01**
SQLE	1.36	1.19	1.56	**< 0.01**
FADS2	1.28	1.17	1.40	**< 0.01**
ABCC1	1.17	0.97	1.41	**0.02**
SLC1A5	1.29	1.09	1.52	**< 0.01**

Bold values indicate statistically significant differences (*p* < 0.05).

### GSVA and ssGSEA Analyses of Ferroptosis Mediation Patterns

**FIGURE 3:**
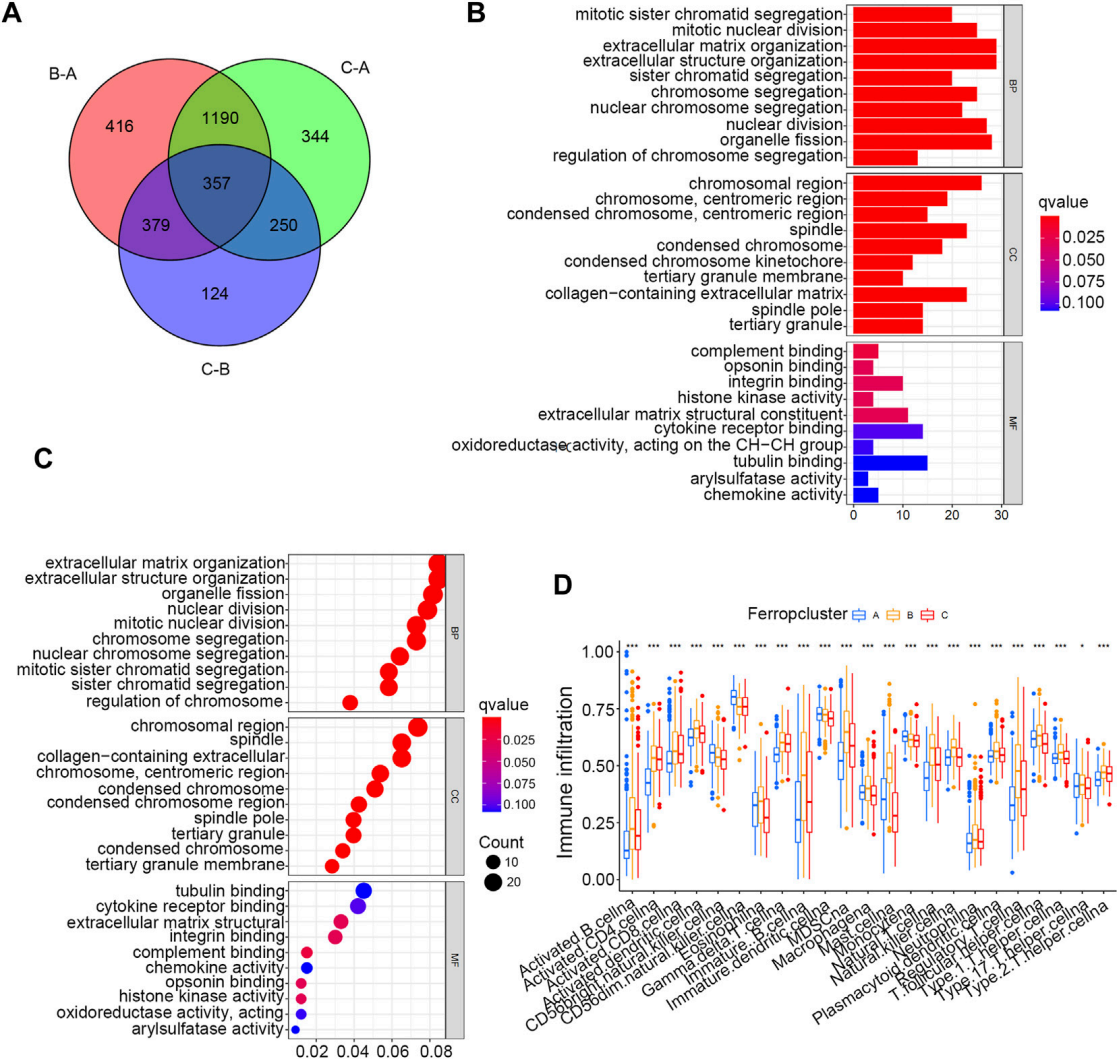
GO and ssGSEA analyses based on DEGs in distinct Ferropclusters. **(A)** 357 DEGs between three distinct Ferropclusters. **(B)** Bar plot presenting GO enrichment analysis. **(C)** Bubble chart presenting GO enrichment analysis. **(D)** ssGSEA analysis of three distinct Ferropclusters. The asterisk symbol indicated the statistical *p*-value. (*p < 0.05; **p < 0.01; ***p < 0.001).

To further discover the potential biological activities underlying different ferroptosis mediation patterns, GSEA was conducted, which revealed that Ferropcluster A was prominently enriched in lipid metabolism activities including glycerophospholipid and arachidonic acid metabolism and various carcinogenic activation processes including the TGF-beta and VEGF signalling pathways (Supplementary Figure S2 and Supplementary Table S7). Glycerophospholipid and arachidonic acid metabolism had a close relationship in inducing ferroptosis, which was consistent with previous studies, and the significantly improved survival outcomes of Ferropcluster A (Supplementary Figure S1B) might benefit from ferroptosis in cancer cells. (Li and Li, 2020). Ferropcluster B was significantly enriched in various stromal activation processes including ECM receptor interaction, NON-like receptor signalling pathway, cysteine and methionine metabolism and chemokine signalling pathway. Ferropcluster C was mainly enriched in the TCA cycle, butanoate metabolism and chlorophyll metabolism. A total of 357 DEGs (Supplementary Table S8) were identified among different ferroptosis mediation patterns, and GO enrichment analysis (Figures 3A–C) revealed that these DEGs were involved in various cell proliferation processes including nuclear division, chromosome segregation and mitotic nuclear division. Furthermore, ssGSEA analysis revealed that ferropcluster A had significantly increased infiltration of innate immune cells including natural killer cells, immature dendritic cells and monocytes, which was consistent with its improved survival status (Figure 3D). However, ferropcluster B had the highest infiltration of various immune cells, which was inconsistent with its poorer prognosis (Supplementary Figure S1B). This result may be attributed to significantly enriched stromal activation in Ferropcluster B, which is responsible for immune suppression. (Chen et al., 2017).

### Construction of a Ferroptosis Genetic Signature

**FIGURE 4:**
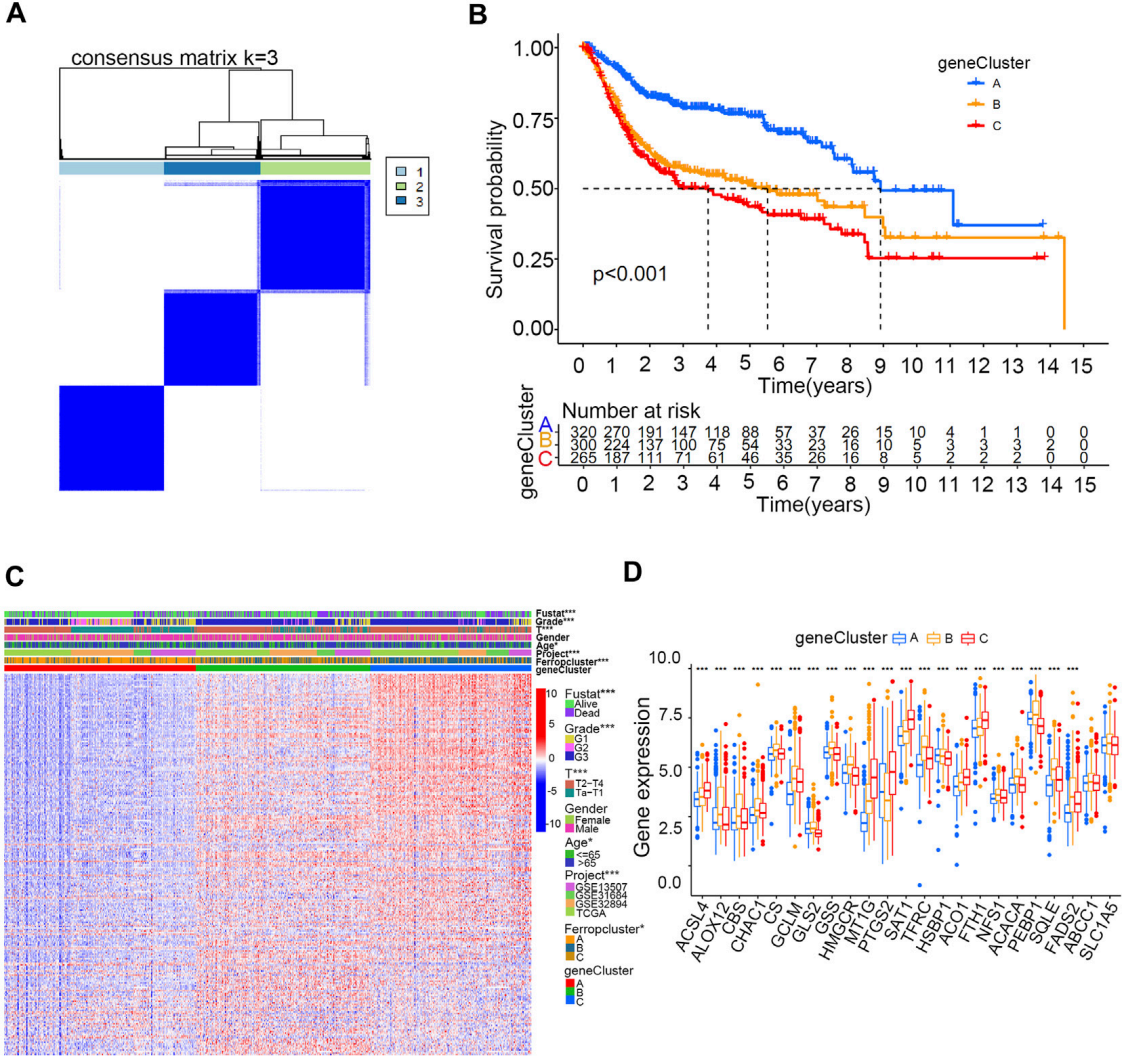
Ferroptosis gene clusters in the merged cohort. **(A)** Consensus clustering matrix for k = 3. **(B)** Kaplan–Meier curve survival analysis among three distinct gene clusters. **(C)** Heatmap demonstrating various clinicopathological features of three distinct m6A clusters. **(D)** Different expression levels of 23 ferroptosis-related genes in distinct gene clusters. The asterisk symbol indicates the statistical *p*-value. (*p < 0.05; **p < 0.01; ***p < 0.001).

**FIGURE 5:**
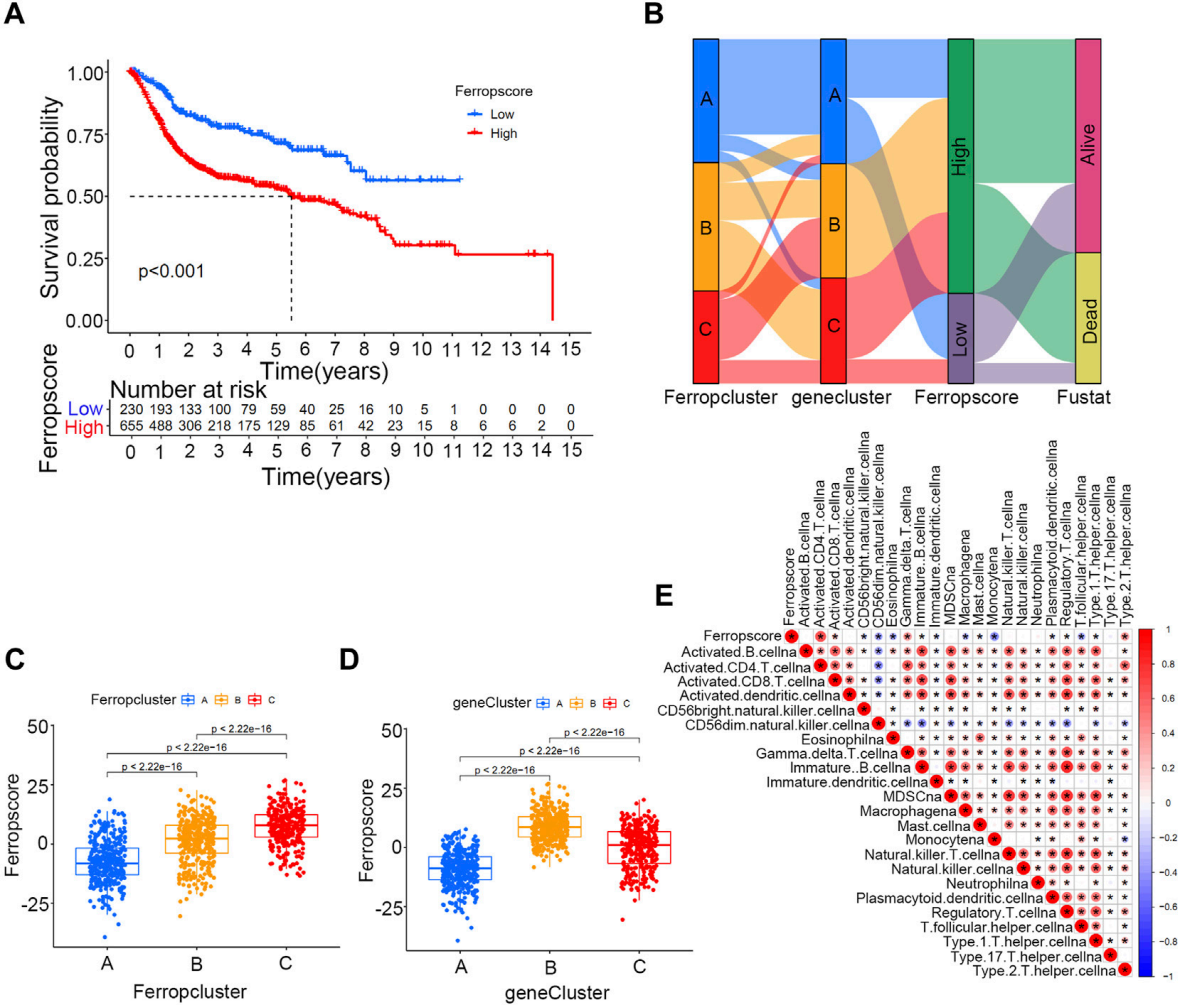
Ferropscore as a quantification tool for individual samples in the merged cohort. **(A)** Kaplan–Meier curve analysis of high/low ferropscore groups. **(B)** Sankey diagram demonstrating correlations among ferropscore, Ferropclusters and ferroptosis gene clusters. **(C)** Differences in ferropscores among three Ferropclusters in the merged cohort. **(D)** Differences in ferropscores among three gene clusters in the merged cohort. **(E)** ssGSEA analysis demonstrating a correlation between ferropscore and infiltration abundance of various immune cells.

Of the 357 DEGs identified among distinct ferroptosis mediation patterns, 251 (70.3%) were found to play a significant role in predicting survival outcomes (Supplementary Table S9 and Supplementary Table S10). Furthermore, unsupervised clustering analyses were conducted based on these prognostic DEGs to further classify the samples into distinct ferroptosis genetic groups. In total, three genetic groups were identified, which were named ferroptosis gene clusters A, B, and C (Figure 4A and Supplementary Table S11). Figure 4B indicates significant differences in survival outcomes among distinct ferroptosis gene clusters, and Figure 4C demonstrates the relationship between the three clusters and various clinical parameters. We discovered that most ferroptosis-related genes in the merged cohort were differentially expressed in distinct gene clusters, excluding ABCC1 and SLC1A5 (Figure 4D). To further quantify ferroptosis mediation patterns in individual samples, ferropscore was developed based on the expression levels of prognostic DEGs using the PCA algorithm (Supplementary Table S12 and Supplementary Figure S13). Figure 5A demonstrates significant differences in survival outcomes between the high and low ferropscore groups (*p* < 0.001). The low ferropscore group had improved survival benefits. Figure 5B demonstrates internal relationships among ferropscore, ferropclusters and ferroptosis gene clusters on a Sankey diagram. As shown in Figures 5C,D, both ferropcluster A (*p* < 0.001) and gene cluster A (*p* < 0.001) have the lowest ferropscore, which is consistent with the results presented in Supplementary Figure S1B and Supplementary Figure S4B. Figure 5E demonstrates a positive correlation between ferropscore and infiltration of activated CD4 T cells, activated CD8 T cells, gammadelta T cells and type 2 T helper cells and a negative correlation between ferropscore and infiltration of CD56 bright natural killer cells, CD56 dim natural killer cells, eosinophils, macrophages, monocytes, T follicular helper cells and regulatory T cells.

### Positive Correlation Between Ferropscore and Tumour Mutation Burden in Bladder Cancer

**FIGURE 6:**
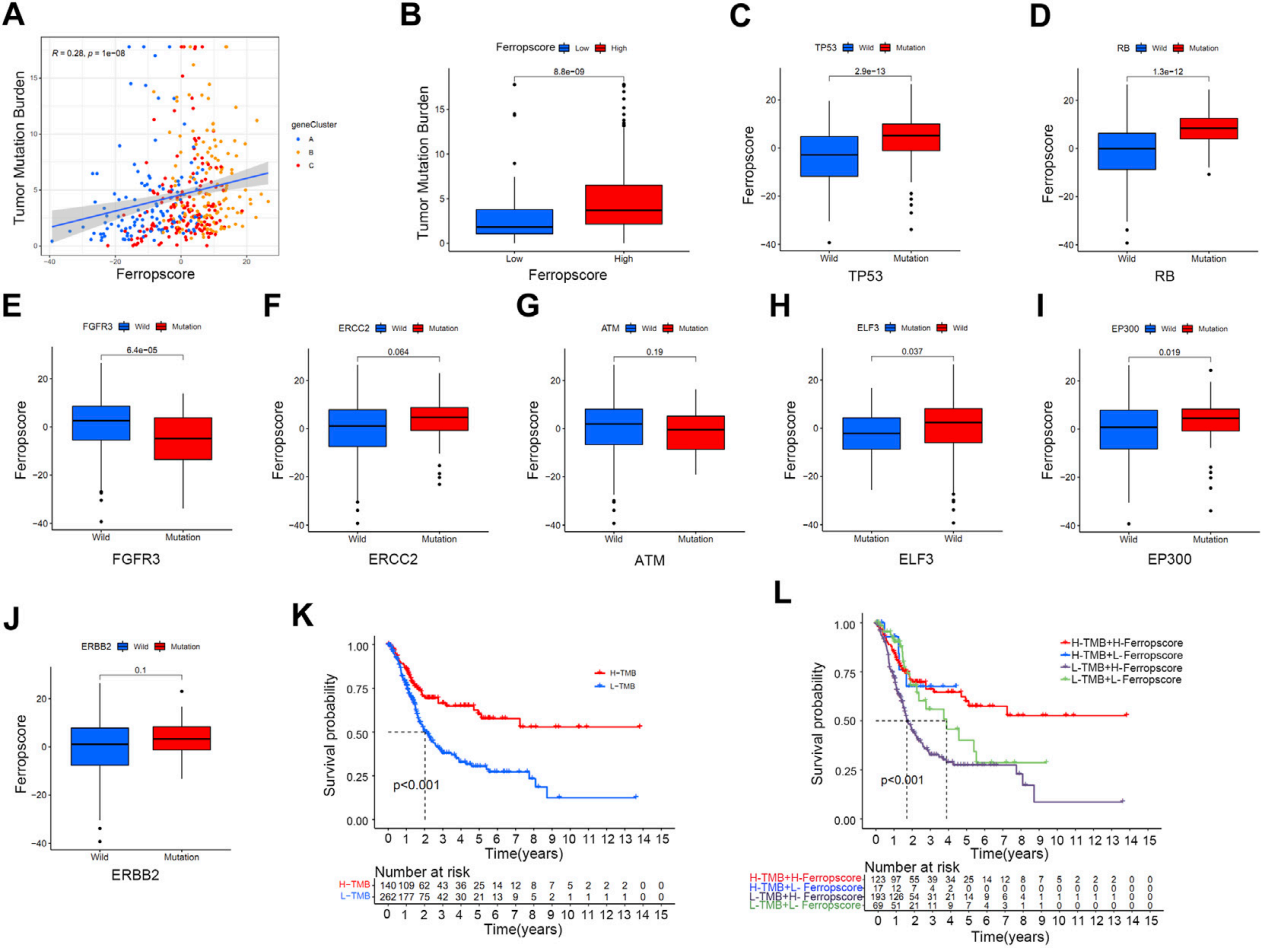
Relationship between ferropscore and tumour genetic mutations. **(A)** Positive correlation between ferropscore and TMB in bladder cancer. **(B)** Differences in the TMB value between the high/low ferropscore groups. **(C–J)** Differences in ferropscores between the wild and mutation status of typical oncogenes including TP53, RB, FGFR3, ERCC2, ATM, ELF3, EP300 and ERBB2. **(K)** Kaplan–Meier curve analysis showing prognosis benefits of high TMB. **(L)** Kaplan–Meier curve analysis concerning the combination of ferropscore and TMB.

Figure 6A demonstrates a positive correlation between ferropscore and TMB in bladder cancer (*p* < 0.001). The high ferropscore group had higher TMB than that of the low ferropscore group (Figure 6B). These results indicated potential interactions between ferroptosis and genetic mutation in bladder cancer; therefore, we further examined the relationship between ferropscore and several typical oncogenic mutations reported to be responsible for bladder cancer. (Liu et al., 2021a). Figure 6 demonstrates that mutated types of TP53 (*p* < 0.001), RB (*p* < 0.001), ELF3 (*p* = 0.037) and EP300 (*p* = 0.019) had significantly higher ferropscore than that of the wild types. Mutations in FGFR3 had significantly lower ferropscore than that of the wild types (*p* = 0.019), whereas no obvious differences were observed in ferropscore between the mutated and wild types of ERCC2, ATM and ERBB2. These results provided novel insights into the relationship between ferroptosis mediation patterns and genetic mutations in bladder cancer. Figure 6K demonstrates improved survival outcomes in the high TMB group, which is consistent with previous studies. (Patel et al., 2020). Figure 6L demonstrates that groups with high TMB and lower ferropscores exhibit the best survival outcomes, whereas groups with low TMB and higher ferropscores exhibit the worst survival outcomes in bladder cancer.

### Relationships Between Ferropscore and Various Clinical Parameters and Molecular Subtypes of Bladder Cancer

**FIGURE 7:**
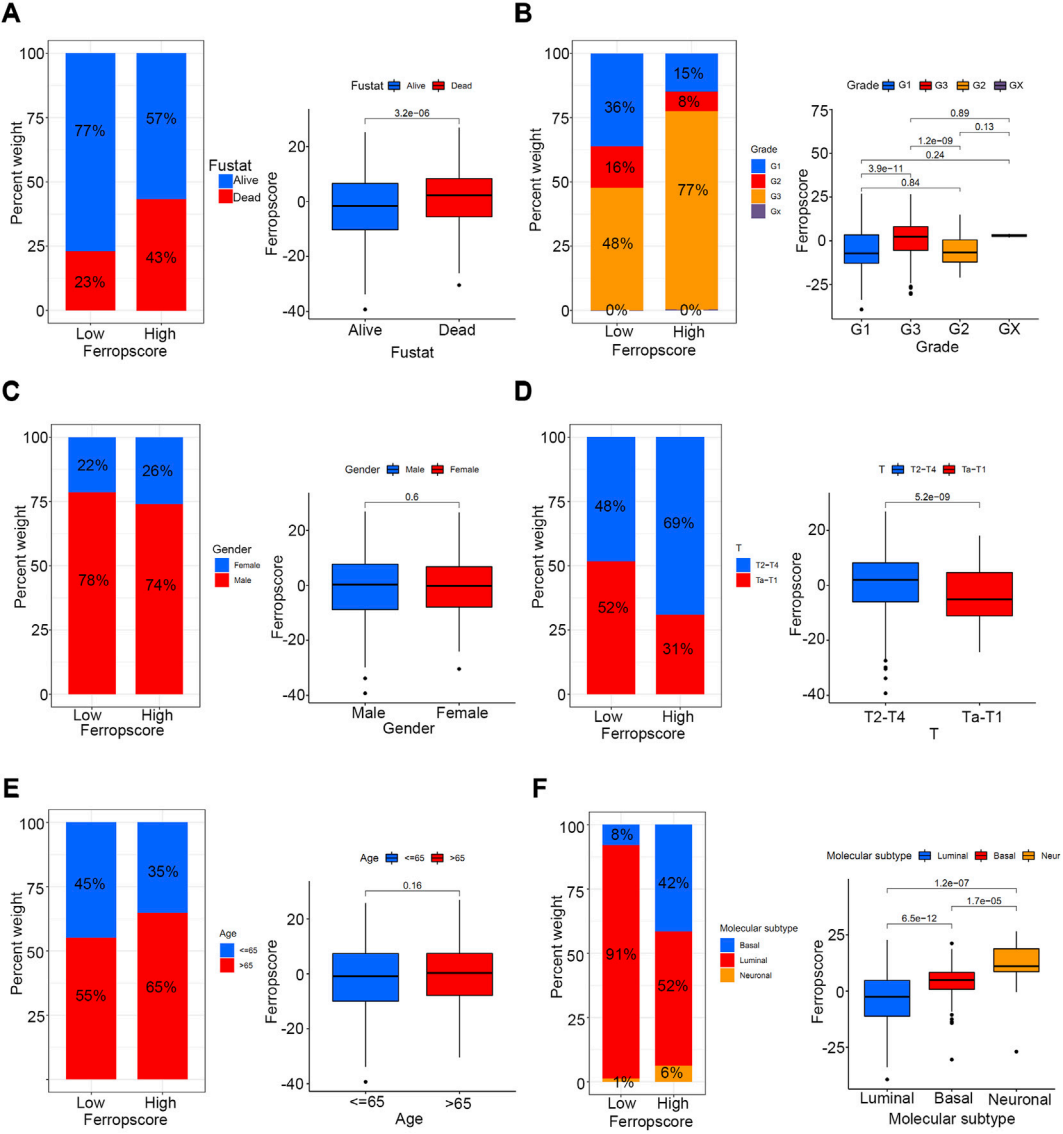
Relationship between ferropscore and various clinical parameters including age, alive/dead status, sex, tumour stages, tumour grades and molecular subtypes of bladder cancer. **(A)** Relationships between ferropscore and alive/dead status. **(B)** Relationship between ferropscore and tumour grades. **(C)** Relationship between ferropscore and sex. **(D)** Relationship between ferropscore and clinical stages. **(E)** Relationship between ferropscore and age. **(F)** Relationship between ferropscore and molecular subtypes.

**FIGURE 8:**
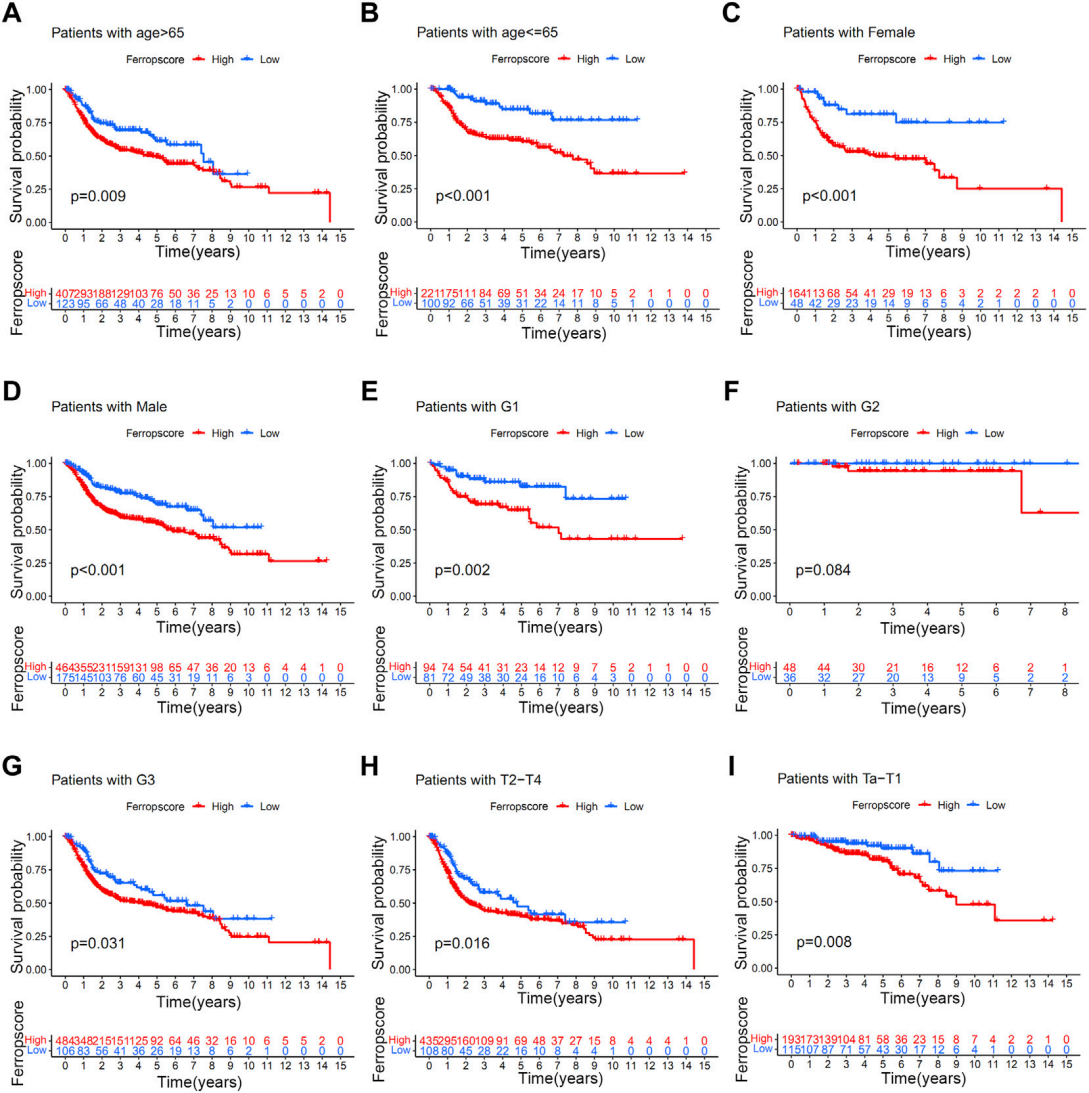
Kaplan–Meier survival analysis of high/low ferropscores in different subgroups of the merged cohort. **(A)** Kaplan–Meier survival analysis in patients aged >65 years. **(B)** Kaplan–Meier survival analysis in patients aged ≤ 65 years. **(C)** Kaplan–Meier survival analysis in female patients. **(D)** Kaplan–Meier survival analysis in male patients. **(E–G)** Kaplan–Meier survival analysis in patients with grade 1, grade 2 and grade 3 disease. **(H)** Kaplan–Meier survival analysis in patients with Ta–T1 stage disease. **(I)** Kaplan–Meier survival analysis in patients with T2–T4 stage disease.

To investigate the potential prediction roles of ferropscore, we further examined the relationship between ferropscore and various clinical parameters (Supplementary Table S14). The molecular subtypes of bladder cancer have been suggested as valuable tools for predicting survival or pathological stages. (Robertson et al., 2017). Figure 7 demonstrates that samples with “dead” status (*p* < 0.001), higher grades (*p* < 0.001) and advanced stages (*p* < 0.001) have significantly higher ferropscores, indicating that ferropscore is a promising biomarker for bladder cancer progression. Figure 7F demonstrates that the ‘basal’ and ‘neuronal’ subtypes have significantly higher ferropscores than that of the ‘luminal’ subtype (*p* < 0.001), thus confirming that ferropscore can also be used to classify distinct molecular subtypes. We further analysed the role of ferropscore in predicting overall survival using a subgroup analysis classified by age, sex, tumour grades and tumour stages. Figure 8 demonstrates that ferropscore plays a significant role in predicting survival outcomes in the following groups: age ≤ 65 years (*p* < 0.001), age >65 years (*p* = 0.009), female (*p* < 0.001), male (*p* < 0.001), G1 (*p* = 0.002), G3 (*p* = 0.031), Ta–T1 (*p* = 0.008) and T2–T4 (*p* = 0.016). These results demonstrated that ferropscore played a prognostic role in different groups of patients with bladder cancer, indicating a necessity for deepening ferroptosis mechanism-related studies on bladder cancer tissues in the future.

### Ferropscore Played a Vital Role in Predicting Immunotherapy Response in Bladder Cancer

**FIGURE 9:**
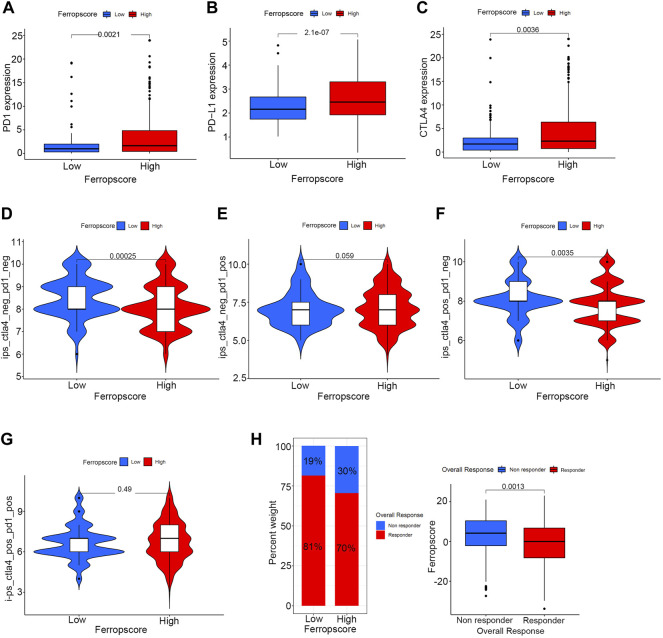
Potential guidance of ferropscore for predicting immunotherapy response in bladder cancer. **(A–C)** Differences in the expression of PD-1, PD-L1 and CTLA-4 between the high and low ferropscore groups. **(D–G)** Differences in the immunotherapeutic effects of four different strategies between the high and low ferropscore groups. **(H)** Evaluation of the role of ferropscore in predicting immunotherapy responses using the TIDE algorithm.

Figure 9A illustrates that the high ferropscore group exhibits higher expression levels of PD-1 (*p* = 0.002), PD-L1 (*p* < 0.001) and CTLA-4 (*p* = 0.004) than those in the low ferropscore group. This indicated that ferropscore might play a role in predicting immunotherapy response in bladder cancer. Furthermore, immunotherapy files were derived from http://tcia.at/and were used to investigate the potential predictive roles of ferropscore in immunotherapy response (Supplementary Table S15). Figure 9 demonstrates that the low ferropscore group exerted significantly improved immunotherapeutic effects when anti-CTLA4 strategies were administered. The immunotherapy responses calculated from the TIDE algorithm were further used to validate relationships between ferropscore and immunotherapy response (Supplementary Table S16). Figure 9H confirms that low ferropscore indicates a better immunotherapy response in bladder cancer. These results confirmed a close relationship between ferroptosis and immunotherapy response in bladder cancer. Furthermore, we also provided all R codes used for data analysis in our study by Supplementary Table S17.

### FADS2 and HMGCR Are Overexpressed in Bladder Cancer Cell Lines

**FIGURE 10:**
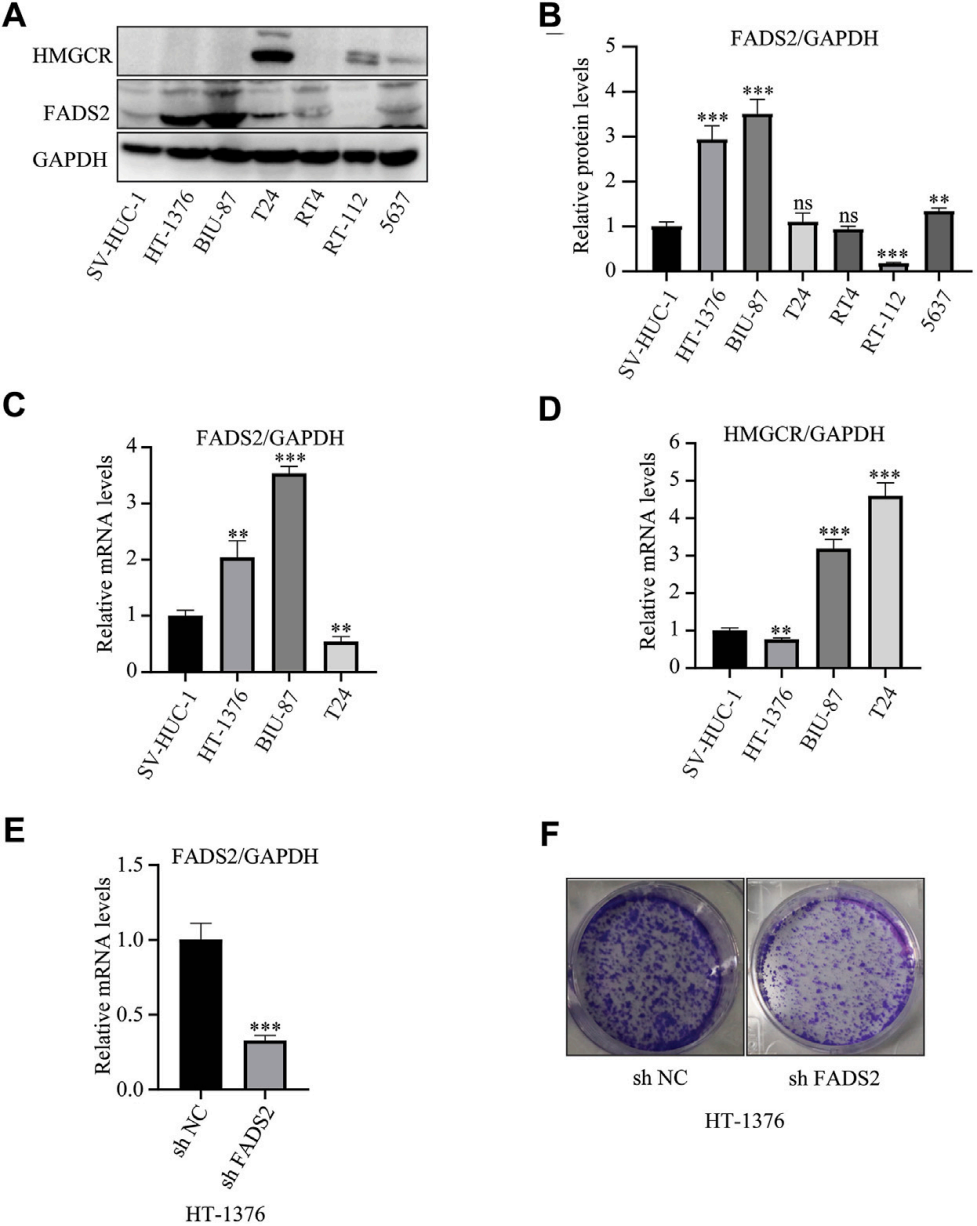
FADS2 and HMGCR expression in bladder cancer cell lines. **(A)** Evaluation of the protein expression levels of FADS2 and HMGCR using western blotting. **(B)** Quantification of the relative FADS2/GAPDH levels using the ImageJ software, normalised with the SV-HUC-1 cell group of each blot. **(C)** Evaluation of the mRNA expression levels of FADS2 in three bladder cancer cell lines and normal SV-HUC-1 cells using quantitative PCR, with the expression levels normalised to those of GAPDH. **(D)** Evaluation of the mRNA expression levels of HMGCR in three bladder cancer cell lines and normal SV-HUC-1 cells using quantitative PCR, with the expression levels normalised to those of GAPDH. **(E)** Evaluation of the mRNA expression levels of FADS2 in HT-1376 cells treated with shFADS2 or shNC. **(F)** Crystal violet staining of HT-1376 cells treated with shFADS2 or shNC for 12–14 days. Experiments were performed in triplicates. All data are expressed as mean ± standard deviation (SD). *p < 0.05, **p < 0.01, ***p < 0.001 vs control group.

Our group had firstly indicated a promising roles of ferroptosis-related genes in bladder cancer development. (Liu et al., 2021d). The previous research successfully constructed a ferroptosis-related signature with the GEO cohort based on four genes-FADS2, FANCD2, HMGCR and ALOX5, and this four gene-signature had also been successfully validated utilizing TCGA cohort. Furthermore, Figure 2 in this study also demonstrated that both FADS2 and HMGCR posed significantly predictive roles for overall survivals in our merged cohort. Thus, we further investigated the protein expression levels of these two ferroptosis-related genes in six bladder cancer cell lines, namely, HT-1376, BIU-87, T24, RT4, RT-112 and 5,637. The results indicated that FADS2 protein was overexpressed in most of these bladder cancer cells except for RT-112, whereas HMGCR was overexpressed in T24, RT-112 and 5,637 cells. Both FADS2 and HMGCR proteins exhibited low expression in the human normal ureteral epithelial cells SV-HUC-1 (Figures 10A,B). Next, we chose HT-1376, BIU-87 and T24 cells for FADS2 and HMGCR mRNA expression levels analysis. The qRT-PCR analysis indicated that the FADS2 mRNA expression levels were higher in HT-1376 and BIU-87 cells than in the human normal ureteral epithelial cells, SV-HUC-1 (Figure 10C). HMGCR mRNA was overexpressed in BIU-87 and T24 cells as compared with SV-HUC-1 cells (Figure 10B). These results indicated that the ferroptosis-related genes FADS2 and HMGCR were generally overexpressed in bladder cancer cell lines, which was consistent with our previous analyses. The gene-specific primers used in the analysis are listed in Table 2. What’s more, to further confirm the function of these ferroptosis-related genes, we chose to knock down the FADS2 gene in HT-1376 cells by shRNA. As is shown in Figures 10E,F, FADS2 knock down suppressed the colony formation activity of HT-1376 cells, indicating the importance of ferroptosis-related gene FADS2 in bladder cancer cells proliferation. Supplementary Figure 3 further provided the typical complete-picture of western blotting in our study.

**TABLE 2:** Primer sequences for real-time RT-PCR.

Segments	Primer sequences (5′ to 3′)
FADS2-F	TGC​AAC​GTG​GAG​CAG​TCC​TTC​T
FADS2-R	GGC​ACA​TAG​AGA​CTT​CAC​CAG​C
HMGCR-F	GAC​GTG​AAC​CTA​TGC​TGG​TCA​G
HMGCR-R	GGT​ATC​TGT​TTC​AGC​CAC​TAA​GG
GAPDH-F	GGT​ATC​GTG​GAA​GGA​CTC​ATG​AC
GAPDH-R	ATG​CCA​GTG​AGC​TTC​CCG​TTC​AG

### Immunohistochemical Staining

**FIGURE 11:**
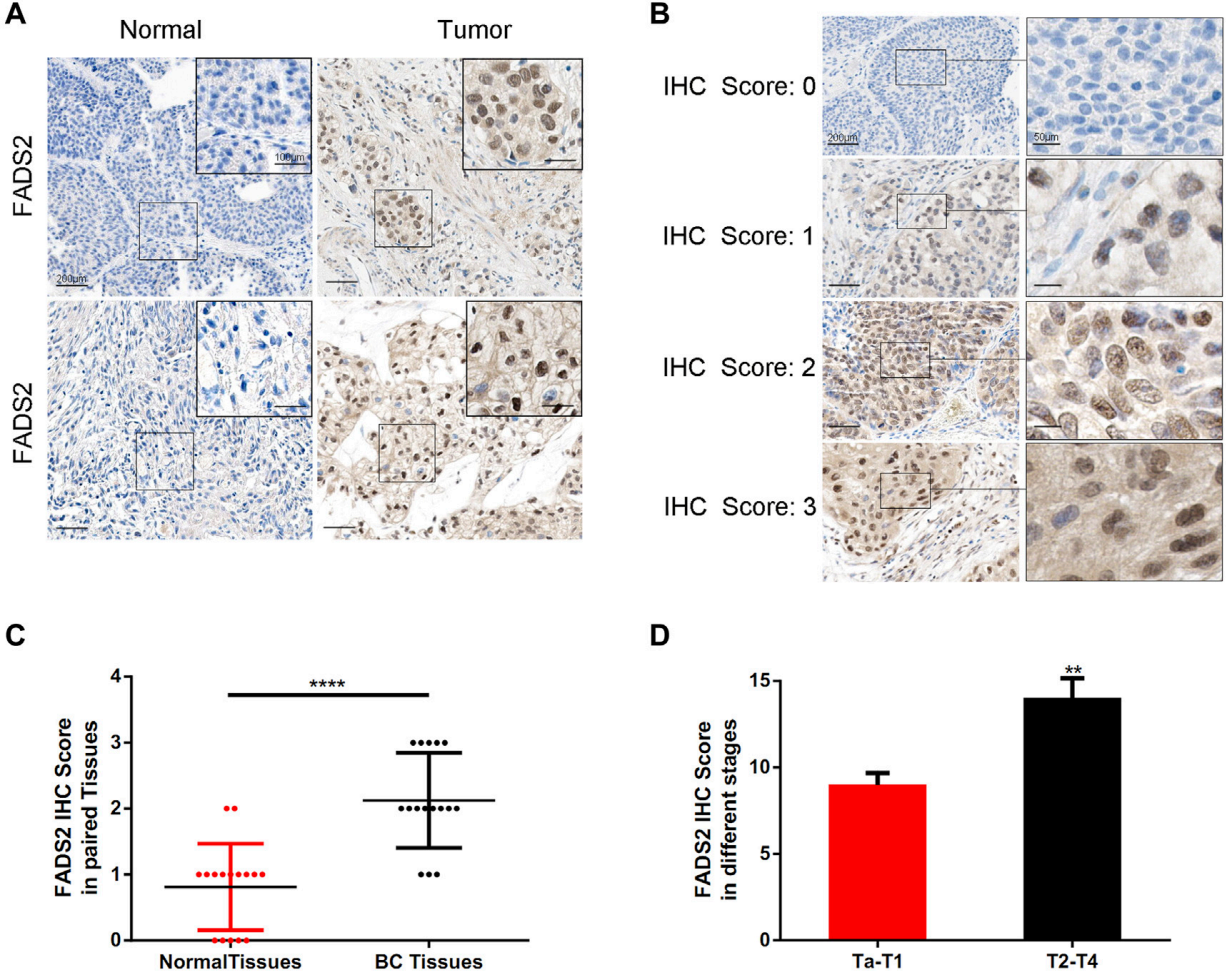
Detection of FADS2 protein expression in tissues by IHC staining. **(A)** IHC of FADS2 in noncancerous tissues and bladder cancer tissues of 2 patients with different tumor grades. The scale bars represent 200 μm and in the inserts 100 μm. **(B)** IHC of FADS2 in bladder cancer tissues. IHC scoring was performed according to the staining intensity (0, negative; 1, weak; 2, moderated; 3, strong). The scale bars represent 200 μm and in the inserts 50 μm. **(C)** FADS2 was significantly increased in primary tumor specimens compared with adjacent non-tumor tissues by IHC. (*p* < 0.0001) **(D)** FADS2 was significantly increased in tumor tissues of T2-T4 stages compared with Ta-T1 stages by IHC. (*p* < 0.01).

Figure 11 demonstrates the typical IHC staining images for FADS2 protein of 16 pairs of bladder tumour tissues. The expression of FADS2 protein was significantly enhanced in tumour tissues as compared with adjacent normal tissues (*p* < 0.001, *n* = 16). Tumours at T2–T4 stages had higher IHC scores than those of tumours at Ta–T1 stages in our cohort (*p* < 0.01). These results indicated that the ferroptosis-related gene FADS2 played a promising role in predicting different bladder tumour stages in clinical settings. Corresponding clinical parameters of IHC samples were also listed in Supplementary Table S14. Detailed studies regarding the mechanism of action of FADS2 in bladder cancer are ongoing at our centre, which will provide a novel perspective for the future treatment of bladder cancer.

## Discussion

According to a recent statistical report, approximately 500,000 patients are diagnosed with bladder cancer annually, indicating bladder cancer as one of the most common malignancies worldwide. (Lenis et al., 2020). Although bladder cancer can be treated surgically, patients may relapse and develop metastasis. For advanced bladder tumours, cisplatin-based chemotherapy is suggested as the first-line treatment and immunotherapy is also currently suggested as the second-line treatment. However, owing to limited knowledge regarding the mechanisms underlying bladder cancer development, the treatment response to chemotherapy or immunotherapy is limited, with high heterogeneity. (Witjes et al., 2021). In 2012, Dixon reported for the first time a novel form of regulated cell death named ferroptosis, which is characterised by iron dependence and lipid peroxide accumulation. (Dixon et al., 2012). Increasing studies in recent years have reported a close relationship between ferroptosis and various malignancies. (Guo et al., 2018; Wang et al., 2019; Dai et al., 2020). In addition, studies have indicated that ferroptosis can serve as a promising candidate for research on malignant cancers resistant to current chemotherapy or immunotherapy. (Bailey et al., 2018; Tsoi et al., 2018). With the rapid development of genetic sequencing techniques, genetic signatures derived from transcriptome information have been reported to predict different treatment responses. (Bailey et al., 2018; Zhang Y et al., 2021; Zhang M et al., 2021). We reported for the first time that ferroptosis-related genes played predictive roles in bladder cancer, and the mechanisms underlying ferroptosis provide promising avenues for developing novel treatment strategies. (Liu et al., 2021a). We previously constructed a ferroptosis-related signature with the GEO cohort based on four genes, including FADS2, FANCD2, HMGCR and ALOX5. (Liu et al., 2021a). However, our previous studies included only 256 bladder cancer samples, a sample size too limited to analyse internal relationships between ferroptosis-related genes and bladder cancer. This study aimed to further expand cancer samples by combining four different cohorts-TCGA, GSE13507, GSE32894, and GSE31684, and to further comprehensively explore whether distinct ferroptosis-mediation patterns existed in bladder cancers. Although only 23 ferroptosis-related genes were identified from these four cohorts due to different sequencing platforms, deeper analysis depending on these intersected genes had also confirmed that, distinct ferroptosis mediation patterns did exist in bladder cancers and quantifying for these patterns demonstrated promising application prospects in predicting multi-omic characteristics as well as distinct immunotherapy responses for bladder cancers.

In total, three distinct ferroptosis mediation patterns were identified in this study, and Ferropcluster A exhibited the best survival outcomes of the three Ferropclusters. The ssGSEA analysis demonstrated that Ferropcluster A had significantly enhanced infiltration of innate immune cells including natural killer cells, immature dendritic cells and monocytes, which was consistent with its improved survival status (Figure 3D). The GSVA analysis demonstrated that Ferropcluster A was prominently enriched in lipid metabolism activities including glycerophospholipid and arachidonic acid metabolism and various carcinogenic activation processes including the TGF-beta and VEGF signalling pathways. Considering these results, we believed that enhanced lipid metabolism in Ferropcluster A increased the accumulation of peroxide products and sensitised cancer cells to ferroptosis, thus suppressing tumour proliferation by activating the innate immune system, leading to a good survival outcome. (Li and Li, 2020; Stockwell and Jiang, 2019). Ferropcluster B had the highest infiltration of various immune cells, which was inconsistent with its poorer prognosis (Supplementary Figure 1B). This may be attributed to enriched stromal activation in Ferropcluster B, which is reported to be responsible for immune suppression. (Gajewski, 2015; Chen and Mellman, 2017). Moreover, although ferropcluster B had an increased abundance of various immune cells, these cells were only confined to the stroma around tumour cells and not into their parenchyma, thus leading to a tumour microenvironment lacking immune cells. (Salmon et al., 2012; Patel et al., 2020; Liu et al., 2021a).

In fact, there were many inconsistencies in referencing ferroptosis-related genes during various ferroptosis-related studies. (Zhou and Bao, 2020; Liu et al., 2021a; Liu et al., 2021e). Liu et al. recently identified two distinct ferroptosis mediation patterns in the hepatocellular carcinoma on basis of 74 ferroptosis-related genes by reviewing relative researches, however, that study did not conduct further experimentally validation and just pointed out a potential direction for future malignancies treatment. (Liu et al., 2021a). Various other studies also explored potential implications of ferroptosis by referring to FerrDb database, (Zhou and Bao, 2020), one database which was dedicated to collecting and collating the potential ferroptosis-related regulators. (Zheng et al., 2021). However, the FerrDb database collected all reported ferroptosis-regulators by searching all published articles between January 2012 and February 2020, but did not set other screening conditions for the included studies, such as impact factors, research qualities, etc. Furthermore, a considerable number of genes related to many benign diseases, such as aggravate degenerative diseases, Huntington’s disease, paralysis and Alzheimer’s disease, had also been included into FerrDb database collections. Our study systematically reviewed high-quality studies on the topic of ferroptosis in the last 5 years to identify ferroptosis-related genes in this analysis. The searched high-quality researches on ferroptosis were defined as novel studies with impact factors >10 points and a total of 60 ferroptosis-related genes were included in this study. (Stockwell et al., 2017; Bersuker et al., 2019; Doll et al., 2019; Hassannia et al., 2019). On basis of these ferroptosis-related genes, this study successfully identified three distinct ferroptosis mediation patterns and further confirmed that quantification of ferroptosis mediation patterns in individual samples might help to improve the understanding of multi-omic characteristics of bladder cancer and further guide future immunotherapy responses.

Figure 2A demonstrated that FADS2, TFRC, GCLM, MTIG and HMGCR acted as key factors in the core of the relational network. This study and our previous research have both indicated vital regulatory roles of FADS2 and HMGCR in bladder cancer development, thus these two ferroptosis-related genes were further verified by experiments in this study. The *in vitro* detection revealed that both mRNA and protein levels of FADS2 and HMGCR were generally increased in bladder cancer cells as compared with normal cells, which was consistent with our analyses from public TCGA and GEO datasets. FADS2 is a vital enzyme involved in lipid metabolism and polyunsaturated fatty acid production; the up-regulated or down-regulated expression levels of FADS2 have been reported to be responsible for inflammation and various diseases including neurological and mental diseases, metabolic disorders and malignant cancers. (Liu and McNamara, 2011; Tosi et al., 2014). The FADS2 gene is located on chromosome 11, and the FADS2 protein consists of 444 amino acids and has a molecular weight of 52.2 kDa. He et al. previously reported that both mRNA and protein levels of FADS2 were significantly improved in melanoma tissues as compared with paracancerous tissues, which was also observed in the present study. (He et al., 2012). They also reported that FADS2 suppression or inhibition was responsible for tumour growth suppression. (He et al., 2012). FADS2 knockdown was also reported to significantly inhibit lung cancer development by increasing the levels of lipid peroxidation products and iron, which are the typical characteristics of ferroptosis. (Jiang et al., 2017). However, the detailed expression patterns of FADS2 in bladder cancer cells have never been investigated through experimental validation. In this study, we confirmed that FADS2 expression was significantly improved in bladder cancer cells. The mechanisms underlying the regulatory role of FADS2 in cancer cells may provide a novel perspective for the future treatment of bladder cancer. In addition, HMGCR is a key enzyme of mevalonic acid synthesis and has a close relationship with ferroptosis occurrence. (Shimada et al., 2016). HMGCR expression directly influences isopentenyl pyrophosphate synthesis, which is important for CoQ 10 metabolism. The metabolic disorder of CoQ 10 production or isopentenyl pyrophosphate promotes mitochondrial oxidative damage, which further leads to ferroptosis in cancer cells. (Yang and Stockwell, 2016). Our study also indicated that HMGCR expression was significantly enhanced in the public TCGA and GEO bladder cancer samples. qPCR and western blotting revealed generally high expression levels of HMGCR in bladder cancer cell lines. Therefore, treatment strategies targeting HMGCR by inducing ferroptosis may be a focus area for our future studies on bladder cancer. Furthermore, Figure 2A indicates that FADS2, HMGCR, TFRC, GCLM and MTIG exhibited significant correlations with each other and might act together through an unknown ferroptotic pathway to regulate bladder cancer development. Further studies concerning the mechanism of novel ferroptotic pathways should be undertaken to discover the relationship between ferroptosis and bladder cancer treatment.

In this study, CNV analysis revealed a significant CNV occurrence frequency in ferroptosis-related genes, and most genes were differentially expressed between tumour and normal tissues. These results implied important roles of ferroptosis mediation patterns in bladder cancer occurrence. Transforming the three ferropclusters to distinct gene clusters was necessary for examining the significance of ferroptosis in the merged cohort. Therefore, three ferropclusters were derived based on 23 ferroptosis-related genes in the merged cohort, whereas distinct gene clusters were developed from DEGs in the three ferropclusters, which also played a significant role in predicting survival outcomes. Of the 23 ferroptosis-related genes, 21 were differentially expressed among the three gene clusters. Examining these gene clusters could help to distinguish the intrinsic characteristics of ferroptosis mediation patterns in bladder cancer. Therefore, the ferropscore was further developed based on the ferroptosis gene clusters to quantify ferroptosis mediation patterns of individual samples. Figure 5A indicates that ferropscore can be used to predict survival outcomes. Figures 5C,D demonstrate that both ferropcluster A and gene cluster A had the lowest ferropscore, which was consistent with the improved survival benefits observed in these clusters. Furthermore, a close relationship was identified between ferropscore and TMB, typical oncogenic mutations, immune cell infiltration abundance, tumour stages and pathological grades, molecular types, expression of immune checkpoint inhibitors and distinct immunotherapy responses. These results indicated that ferroptosis played a vital role in bladder cancer occurrence and might be a valuable tool for evaluating patient prognosis from a genetic viewpoint in the future.

Our study demonstrated a positive correlation between ferropscore and infiltration of activated CD4 and CD8 T cells, which were associated with immune system activation. However, ferropscore was negatively correlated with infiltration of CD56 bright natural killer cells, CD56 dim natural killer cells, T follicular helper cells and regulatory T cells, which are associated with immune suppression. Recent studies have reported that immunotherapy can significantly enhance CD8^+^ T cell activation in cancer cells. (Khalil et al., 2016; Zou et al., 2016). The mechanisms underlying the antitumour effects of CD8^+^ T cells have been reported to benefit from enhanced ferroptosis in tumour cells. (Wang et al., 2019). The glutamate–cystine antiporter system xc^−^ mainly suppresses ferroptosis. CD8^+^ cells activated by immunotherapy may significantly down-regulate the SLC7A11 and SLC3A2 expression levels, which are vital components of the glutamate–cystine antiporter system xc^−^. Enhanced ferroptosis in tumour cells may further improve immunotherapeutic effects. Therefore, combination strategies of ferroptosis inducers and immunotherapy provide a novel direction for bladder cancer treatment in the future and may also help to overcome resistance to chemotherapy and immunotherapy.

This study demonstrated improved survival outcomes in the high TMB group, which is consistent with previous studies. (Patel et al., 2020). Groups with high TMB and lower ferropscores had the best survival outcomes, whereas groups with low TMB and higher ferropscores had the worst survival outcomes. Furthermore, ferropscore was positively correlated with TMB in bladder cancer samples (*p* < 0.001). Therefore, a combination of TMB and ferropscore could significantly improve the prediction of overall survival in bladder cancer. Bladder cancer is one of the most common malignancies with high TMB status, and understanding the mechanisms underlying the benefits of high TMB is important. (Glaser et al., 2017). Previous studies have reported that high TMB status can significantly increase the neoantigen burden in tumour cells, which can further enhance the immunotherapeutic effects. (Bellmunt et al., 2017; Jiang et al., 2019). Therefore, the positive correlation between TMB and ferropscore may provide insights into the underlying mechanisms of survival benefit from high TMB. Cells with high TMB are easily monitored by various immune cells and further become sensitive to ferroptosis induced by immunotherapy in bladder cancer. This study further identified that ferropscore had significant relationships with oncogenic mutations including TP53 (*p* < 0.001), RB (*p* < 0.001), ELF3 (*p* = 0.037), EP300 (*p* = 0.019) and FGFR3 (*p* = 0.019) mutations. These results indicated a relationship between ferroptosis and TMB. Furthermore, increasing studies have suggested that molecular subtypes should be used to classify patients with distinct prognoses and stages. These molecular subtypes include “luminal”, “basal” and “neuronal”, and the “luminal” subtype is associated with the best survival prognosis. (Choi et al., 2014; Robertson et al., 2018). In this study, we found that the “basal” and “neuronal” subtypes had significantly higher ferropscore than that of the “luminal” subtype (*p* < 0.001), indicating that ferropscore could also be used to classify distinct molecular subtypes. Therefore, both ferropscore and molecular subtypes can be used to evaluate tumour characteristics from a genetic aspect, which may improve the evaluation accuracy with the rapid development of transcriptome sequencing technology.

Furthermore, we examined the potential prognostic role of ferropscore in predicting overall survival in different subgroups. Ferropscore played a significant role in predicting survival outcomes in the following groups: age ≤ 65 years (*p* < 0.001), age >65 years (*p* = 0.009), female (*p* < 0.001), male (*p* < 0.001) groups, G1 (*p* = 0.002) G3 (*p* = 0.031), Ta–T1 (*p* = 0.008) and T2–T4 (*p* = 0.016). These results further confirmed ferropscore to be a promising biomarker. Furthermore, Figure 9 demonstrates that ferropscore plays a role in predicting the expression levels of immune checkpoint inhibitors and can also provide guidance for predicting immunotherapy response. Patients who did not respond to immunotherapy had higher ferropscores in this study. Similar results have been confirmed by previous studies, which reported that radiotherapy and immunotherapy could enhance lipid oxidation and induce ferroptosis by suppressing SLC7A11. (Lang et al., 2019). High ferropscores indicated ferroptosis tolerance, leading to a poor response to radiotherapy or immunotherapy. With the help of sequencing technology, ferropscore in our study can be used to predict the ferroptosis-sensitive or -tolerant status and further provide guidance in developing treatment strategies for bladder cancer. In conclusion, our study provided novel insights into developing novel combined drug strategies and immunotherapy methods for treating bladder cancer in the future.

The present study also had several limitations. Firstly, the ferroptosis mediation patterns were initially analysed using public cohorts including TCGA and GEO. Further external validation using multi-centre cohorts was required to validate the distinct ferroptosis mediation patterns in bladder cancer. Secondly, totally 23 ferroptosis-related genes were identified in the merged cohort and other potentially-valuable genes might have been overlooked due to different platforms in the four cohorts. However, our analysis depending on these 23 genes have confirmed that distinct ferroptosis mediation patterns did exist in bladder cancers and quantifying for these patterns demonstrated excellent prospects for predicting multi-omic characteristics of bladder cancers. Moreover, studies regarding the detailed mechanisms underlying the regulation of bladder cancer development by ferroptosis-related genes are warranted to overcome chemoresistance or immunotherapy resistance in bladder cancer.

## Conclusion

This study discovered distinct ferroptosis mediation patterns in bladder cancer. The evaluation of these patterns may help to enhance our understanding of various characteristics of bladder cancer and to further provide guidance for immunotherapy. This study also provided a novel prospect for exploring new combined drug strategies or novel immunotherapy methods for bladder cancer treatment.

## Data Availability

The datasets presented in this study can be found in online repositories. The names of the repository/repositories and accession number(s) can be found in the article/Supplementary Material.

## Abbreviations

TCGA, The Cancer Genome Atlas; GEO, Gene Expression Omnibus; KEGG, Kyoto Encyclopedia of Genes and Genomes; GO, Gene Ontology; PD-1, Immune Checkpoint Programmed Cell Death 1; ssGSEA, Single-sample Gene Set Enrichment Analysis; PD-L1, Immune Checkpoint Programmed Cell Death 1 Ligand; DEGs, Differentially Expressed Genes; CTLA-4, Cytotoxic T Lymphocyte Antigen 4; RB1, retinoblastoma 1; TP53, Tumor Protein 53; ERBB2, erb-b2 receptor tyrosine kinase 2; ERCC2, ERCC excision repair; FGFR3, Fibroblast Growth Factor Receptor 3; GSVA, Gene Set Variation Analysis; TMB, Tumour Mutation Burden; CNV, Copy Number Variations; PCA, Principal Component Analysis; IHC, Immunohistochemical.
