# Supplementary figures and images for: Ferroptosis Mediation Patterns Reveal Novel Tool to Implicate Immunotherapy and Multi-Omics Characteristics in Bladder Cancer
Source: Front Cell Dev Biol. 2022 Jan 25;10:791630. doi: 10.3389/fcell.2022.791630 (PMC8821925; doi:10.3389/fcell.2022.791630)

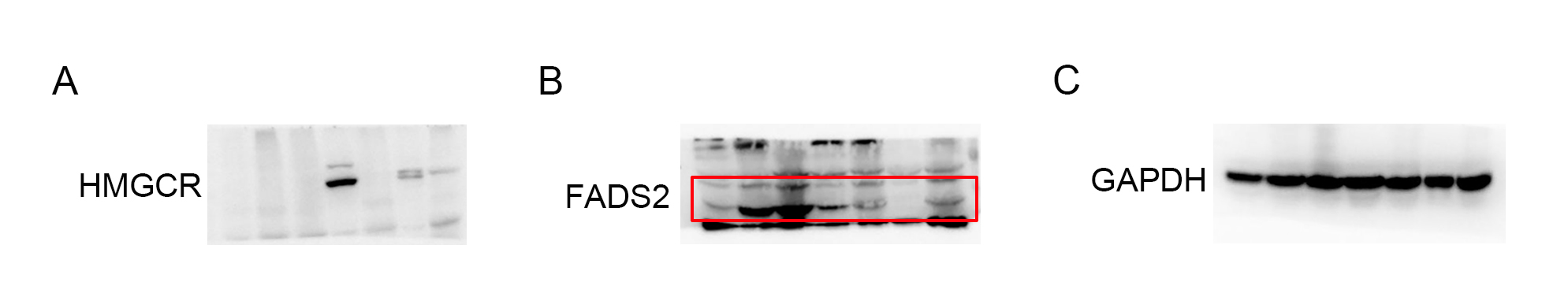

Supplement: Supplementary file 1 [file Image3.JPEG]

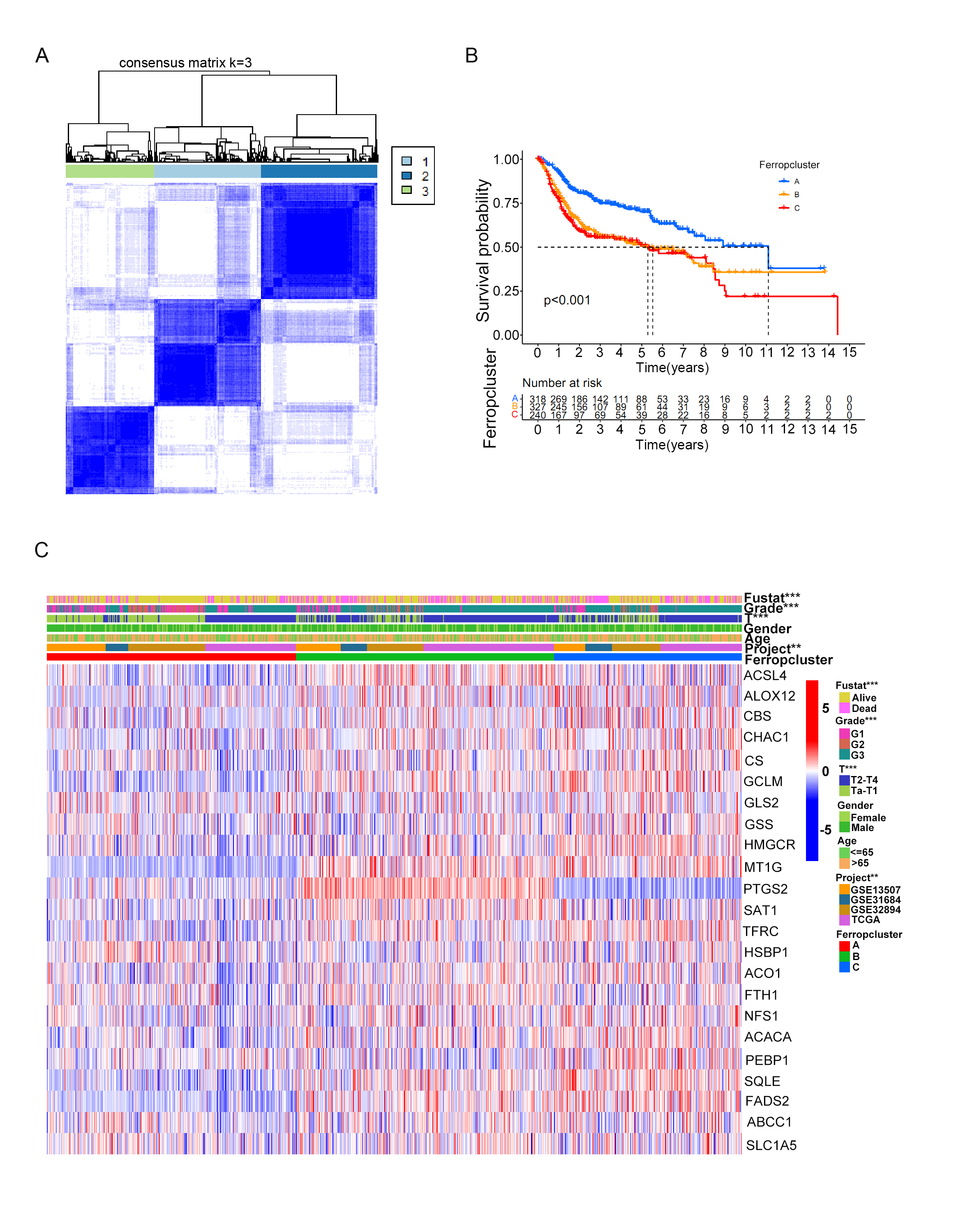

Supplement: Supplementary file 2 [file Image1.JPEG]

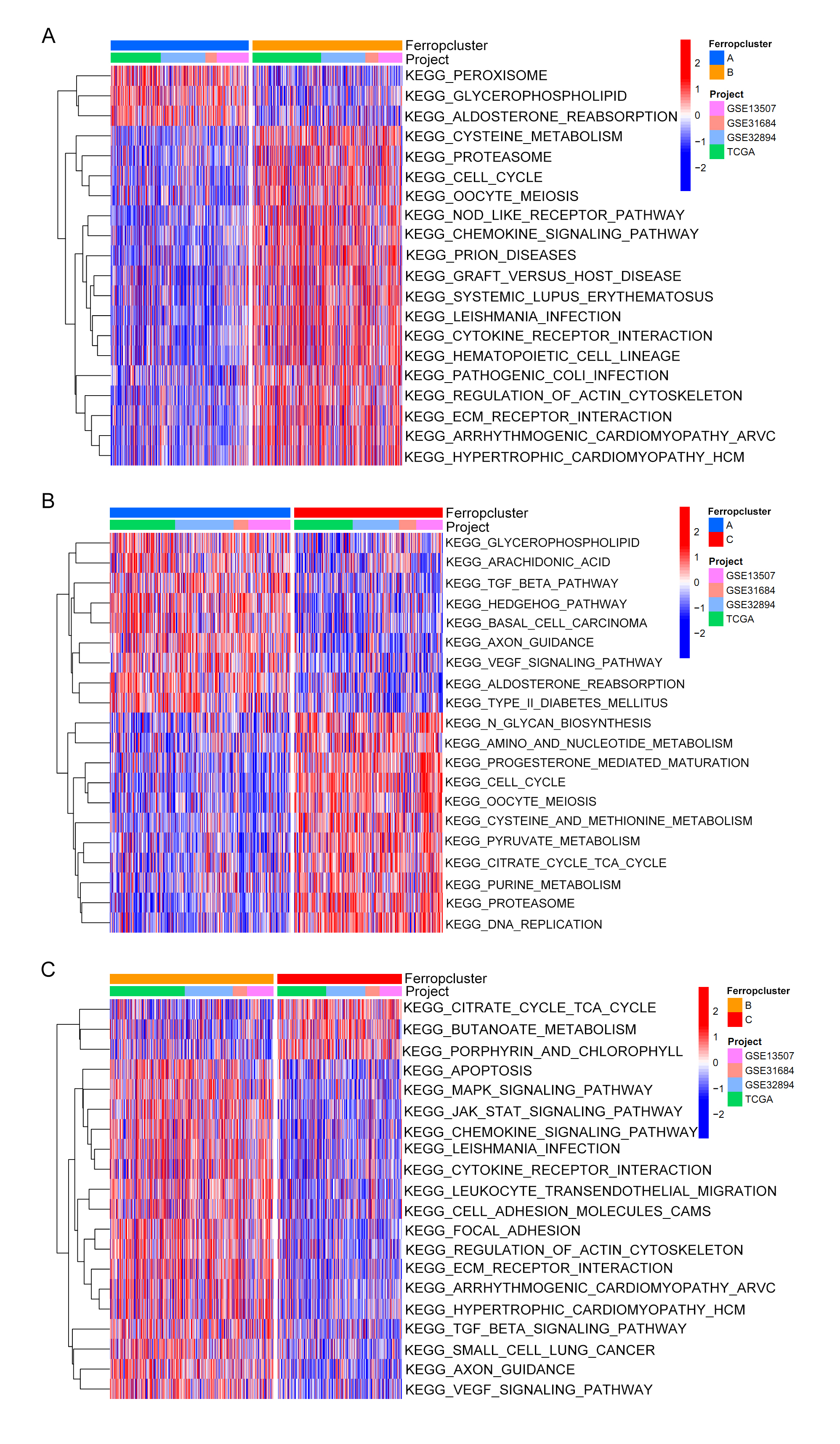

Supplement: Supplementary file 4 [file Image2.JPEG]
